# Outcomes of segmental femoral artery pseudoaneurysm in patients with Behçet’s disease: a single center’s experience

**DOI:** 10.1590/1677-5449.180139

**Published:** 2019-11-05

**Authors:** Hussein Mahmoud Khairy, Ahmed Alaa Shaker

**Affiliations:** 1 Cairo University, Kasr Al Ainy Hospitals, Vascular and Endovascular Department, Cairo, Egypt

**Keywords:** Behçet’s disease, pseudoaneurysm, femoral artery, arteritis, Doença de Behçet, pseudoaneurisma, artéria femoral, arterite

## Abstract

**Background:**

Behçet's disease (BD) is an autoimmune condition that involves multiple systems. The most common arterial manifestation in BD patients is pseudoaneurysm, which has higher frequency than aneurysm formation.

**Objectives:**

To clarify the importance of profunda femoris artery in BD pseudoaneurysm, and present a new method for identifying healthy segments for anastomosis.

**Methods:**

Fifteen patients presented at a vascular department with pseudoaneurysms of the common femoral (CFA) and superficial femoral artery (SFA), were diagnosed with BD and underwent surgical intervention at Kasr al Ainy hospitals over 2 years.

**Results:**

All patients were male. The patients ranged in age from 30 to 40 years (mean, 33.8±2.6 years). Mean duration of the disease was 5±3.4 years. Eight (53.3%) CFA graft interpositions, six (40%) mid SFA graft interpositions, and one (6.66) distal SFA graft interposition were performed. Eight (53.3%) of these operations were performed using Dacron graft and sartorius flap, three (20%) were performed with a polytetrafluoroethylene graft, and four (26.66) with a great saphenous vein graft. Two cases (13.33%) were accidentally discovered while four cases (26.66%) had short distance claudication, four cases (26.66%) had pain at rest, and five cases (33.33%) presented with pulsatile swelling correlated with pseudoaneurysm size (p = 0.005). Patients were followed-up over 1 year for new aneurysms and recurrence.

**Conclusions:**

Surgical repair with muscle flap coverage, with or without ligation of profunda femoris artery, does not affect prognosis. An alternative method for identifying healthy segments of femoral artery may be frozen section and examination of the artery to decrease the risk of recurrence at the site of anastomosis.

## RESUMO

**Contexto:**

A doença de Behçet (DB) é uma enfermidade autoimune que envolve múltiplos sistemas. A manifestação arterial mais comum em pacientes com DB é o pseudoaneurisma, que tem uma frequência mais alta do que a formação de aneurismas.

**Objetivos:**

Esclarecer a importância da artéria femoral profunda no pseudoaneurisma por DB e apresentar um novo método para a identificação de segmentos saudáveis para anastomose.

**Métodos:**

Quinze pacientes se apresentaram em um departamento vascular com pseudoaneurismas da artéria femoral comum (AFC) e artéria femoral superficial (AFS), foram diagnosticados com DB e foram submetidos a intervenção cirúrgica em hospitais Kasr al Ainy ao longo de 2 anos.

**Resultados:**

Todos os pacientes eram do sexo masculino. A idade variou de 30 a 40 anos (média, 33,8±2,6 anos). A duração média da doença foi de 5±3,4 anos. Foram realizadas oito (53,3%) interposições de enxerto na AFC, seis (40%) interposições de enxerto na AFS média, e uma (6.66) interposição de enxerto na AFS distal. Oito (53,3%) das cirurgias foram realizadas utilizando enxertos de Dacron e retalho sartório, três (20%) com enxertos de politetrafluoroetileno, e quatro (26,66) com enxertos da veia safena magna. Dois casos (13,33%) foram descobertos ao acaso, ao passo que quatro casos (26,66%) apresentaram claudicação em curta distância, quatro (26,66%) apresentaram dor em repouso, e cinco casos (33,33%) apresentaram com inchaço pulsátil correlacionado com o tamanho do pseudoaneurisma (p = 0.005). Os pacientes foram seguidos por 1 ano para novos aneurismas e recorrência.

**Conclusões:**

O reparo cirúrgico com cobertura de retalho muscular, com ou sem ligadura da artéria femoral profunda, não afeta o prognóstico. Um método alternativo para identificar segmentos saudáveis da artéria femoral pode ser o exame de congelação e a avaliação da artéria para diminuir o risco de recorrência no local da anastomose.

## INTRODUCTION

Hulusi Behçet described Behçet's disease (BD) as an autoimmune condition that involves multiple systems in 1937.^1^ Diagnostic features of the disease include recurrent oral ulcers, skin and eye involvement, and genital aphthosis. It can affect variable sized segments of both arterial and venous vessels.^2^ Arterial involvement occurs in about 2.2-18% of patients, with a mostly male population.^3^ Vascular involvement occurs as true aneurysm or pseudoaneurysm formation in arteries, thrombotic occlusion in arteries, and thrombosis of veins.^4^ Pseudoaneurysms are seen with a notably higher frequency than true aneurysms in BD patients.^5^

Arteritis, mainly around the vasa vasorum, leads to transmural necrosis, gradual vessel wall thickening, aneurysmal dilatation, pseudoaneurysms, and perforation of the vessel wall, denoting activity.^5^ Pseudoaneurysms are more highly prone to rupture than aneurysms and can be a cause of death in BD patients, due to bleeding or ischemia.^6^

Our aim is to clarify the importance of the profunda femoris artery in BD pseudoaneurysm and the need for a new method to assess healthy segments for anastomosis.

## PATIENTS AND METHODS

Fifteen patients presented to a vascular department with common femoral (CFA) and superficial femoral artery (SFA) pseudoaneurysms, were diagnosed with BD, and underwent surgical intervention at Kasr al Ainy hospitals.

The disease was diagnosed using the clinical criteria defined by the International Study Group for Behçet’s Disease clinical assessment (Table 1),^7^ with diagnostic assistance from the Rheumatology department at our institution.

**Table 1:** Diagnostic criteria for Behçet's disease*.

**Major symptoms**	**Minor symptoms**
Recurrent genital ulcerations	Gastrointestinal features
Aphthous ulceration or scarring	Arthritis
Eye lesions	Family history
Skin lesions	Arthralgia
Positive pathergy test	Cerebral nervous system involvement
	Arterial occlusion or aneurysms
	Epididymitis
	Deep vein thrombosis
	Subcutaneous phlebitis

* Recurrent oral ulceration; minor aphthous, major aphthous, or herpeti-form ulceration observed by physician or patient, recurring at least three times in one 12-month period; and two of the symptoms in the table.

Work up was as follows:

- Full laboratory studies from complete blood count and biochemical analysis with emphasis on erythrocyte sedimentation rate (ESR) > (15 mm/h) and C-reactive protein (CRP) > (5 mg/L), as markers of activity which, if elevated, indicates that immunosuppressive therapy is necessary to induce remission (glucocorticoids at a dose of 2 mg per kg per day for 15 days);
- Imaging investigations in the form of Doppler study and computed tomography angiography (CTA) were done for all patients to plan treatment. All patients with an aneurysm were further examined with computed tomography pulmonary angiography to exclude any concomitant extremity aneurysm and underwent fundus examination;
- Patients gave consent for the surgical intervention with its risks and in accordance with the Helsinki Declaration and in compliance with local ethical guidelines. Ethics committee approval was obtained.

After hospital discharge, all patients were followed-up regularly at 3-month intervals. They were examined in the outpatients clinic for graft patency and formation of new false aneurysms, bleeding, or infection and, in case of suspicion, a further examination was performed, usually CTA.

### Statistical analysis

SPSS version 24 for windows was used for statistical analysis. P values less than 0.05 were considered statistically significant.

## RESULTS

Fifteen arterial aneurysms were diagnosed in 15 patients during the study period. All patients were male. Patient age ranged from 30 to 40 years (mean, 33.8±2.6 years). Mean duration of the disease was 5±3.4 years. Other clinical manifestations are given in Table 2. We found positive family history in 6 patients.

**Table 2:** Other clinical manifestations among patients.

**Manifestation**	**n (%)**
Deep venous thrombosis	4 (26.6%)
Associated iliac block	2 (13.3%)
Associated femoral block	3 (20%*)*

Eight cases (53.3%) had common femoral artery graft interpositions, six cases (40%) had mid superficial femoral artery graft interpositions and one case (6.66) had distal superficial femoral artery graft interposition. Eight (53.3%) of these operations were performed using Dacron graft and sartorius flap, three (20%) were performed with a polytetrafluoroethylene graft, and four (26.66) with a great saphenous vein graft.

During surgery, we performed proximal and distal anastomoses to arteries free from disease and in healthy tissue, judged by the naked eye method. Identification of a non-diseased segment of the arterial tree is not always easy; inspection and palpation were helpful for assessment, but were not accurate.

**Figure 1:**
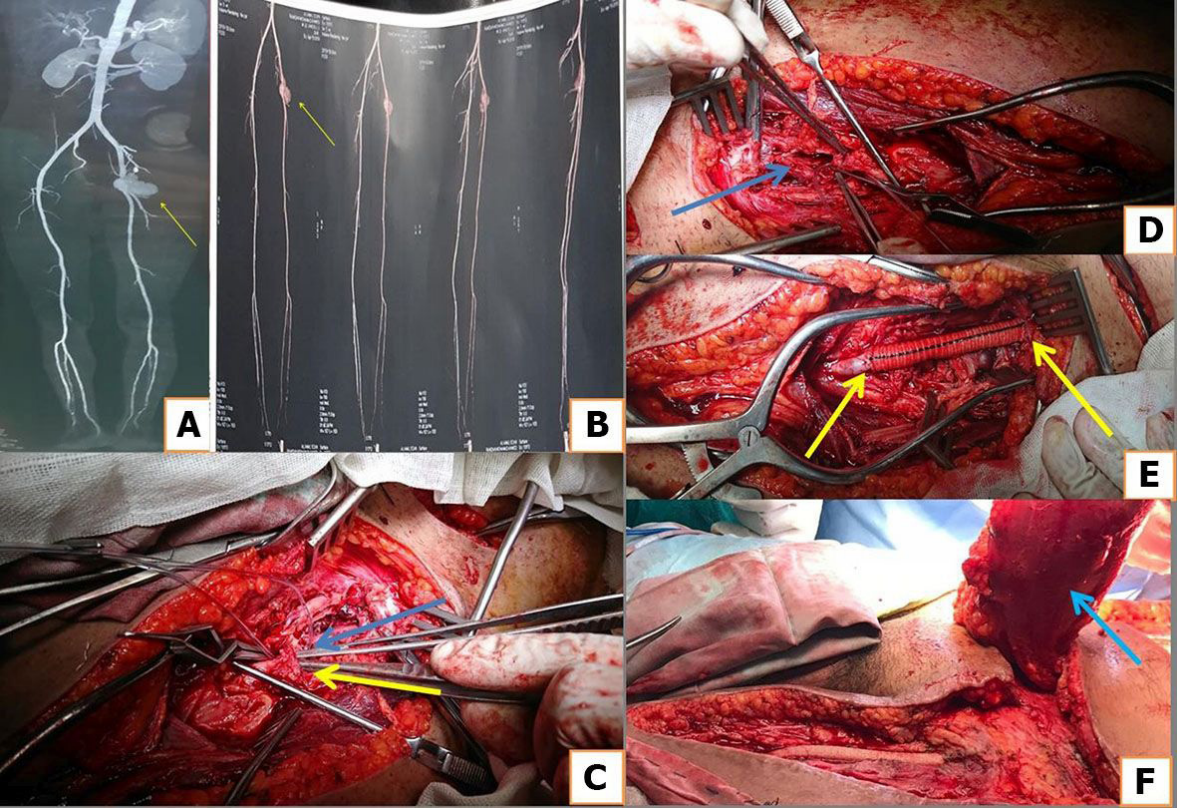
(A, B) Yellow arrow indicates Behçet's pseudoaneurysm of the left common femoral artery, involving the origin of profunda femoris artery, shown by computed tomography angiography (CTA); (C) intraoperative view; blue arrow indicates origin of the profunda femoris artery involved in the pseudoaneurysm; yellow arrow indicates origin of the superficial femoral artery; (D) blue arrow indicates pseudoaneurysm opened after proximal and distal control; (E) yellow arrows indicate proximal and distal anastomoses with 8x60 cm Dacron graft and ligation of the distal profunda femoris artery; (F) blue arrow indicates rectus abdominis flap from contralateral side after 3 secondary hemorrhages and repairs with extension of proximal and distal landings to apparently healthy non diseased artery.

Patients were followed up for an average duration of 4.7 months (range, 3-6 months). Three patients were lost to follow-up, and there was no remote mortality during the follow-up period. One patient returned three times with secondary bleeding: the first time from the ligated distal profunda femoris artery, the second time from the proximal anastomosis, and the third time from the distal anastomosis. The repair was effected by extending proximal and distal anastomoses into more healthy segments and then covered with a contralateral rectus abdominis flap for the groin by the plastic team and doing well (Figure 1).

### Presentation

**Figure 2:**
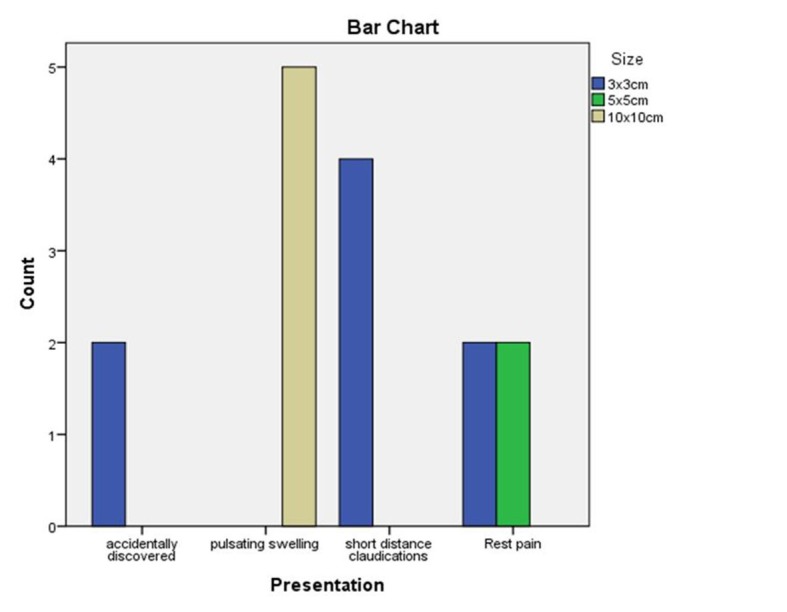
Correlation between patient presentations and pseudoaneurysm size.

Two cases (13.33%) were accidentally discovered, four cases (26.66%) had short distance claudication, four cases (26.66%) had rest pain, and five cases (33.33%) presented with pulsatile swelling correlated with pseudoaneurysm size, as shown in Figure 2, with p = 0.005.

Associated DVT and occlusive disease

**Figure 3:**
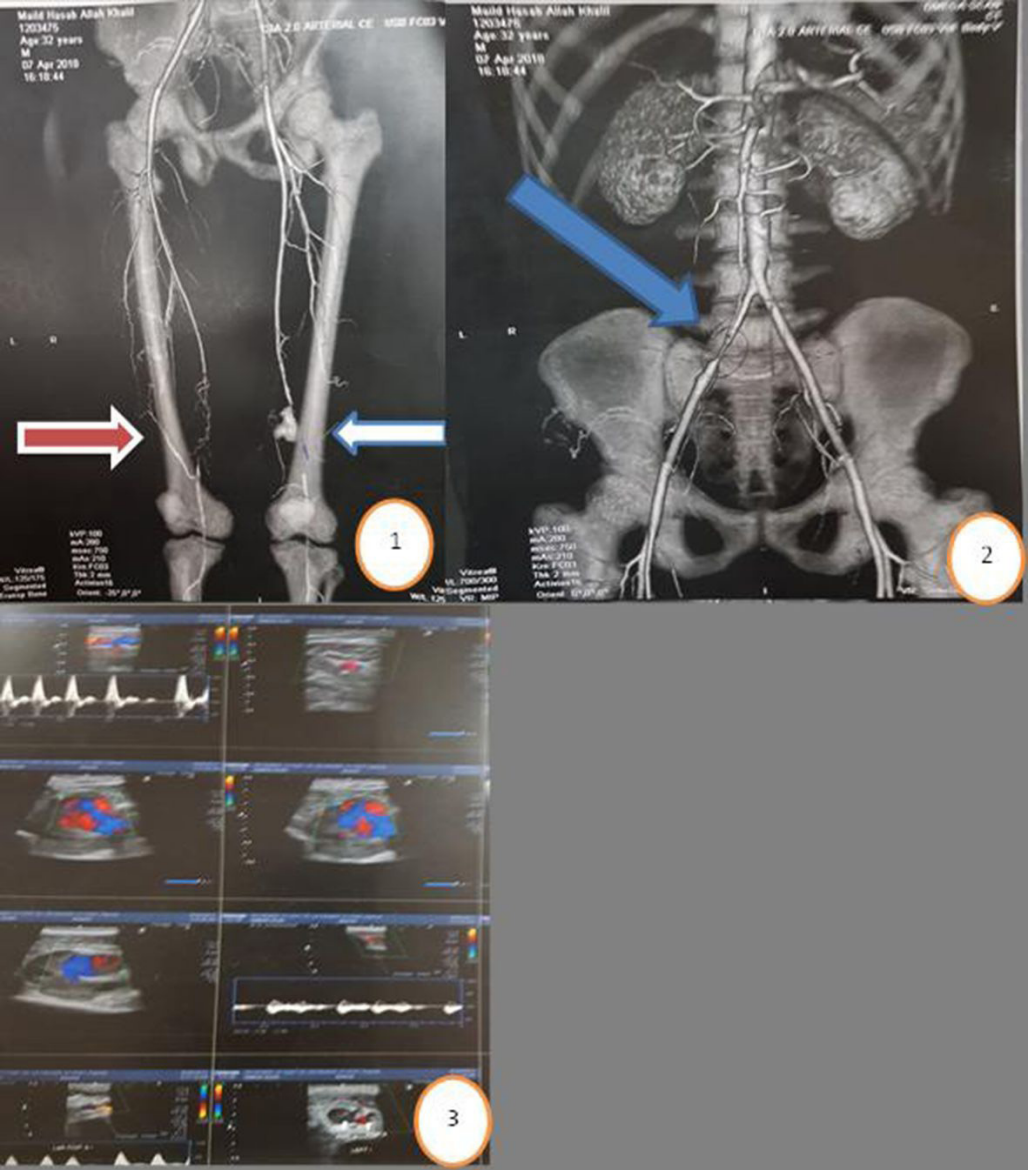
Circle 1: CTA with blue arrow indicating Behçet's pseudoaneurysm of distal left SFA; red arrow indicates associated right SFA block; circle 2: blue arrow indicates associated right iliac occlusion in the same patient, circle 3: Duplex US of left SFA showing pseudoaneurysm.

There was no correlation between age and associated deep venous thrombosis, or between subacute or acute DVT and iliac occlusion or contralateral superficial femoral artery occlusion (Figure 3).

### Procedure

**Figure 4:**
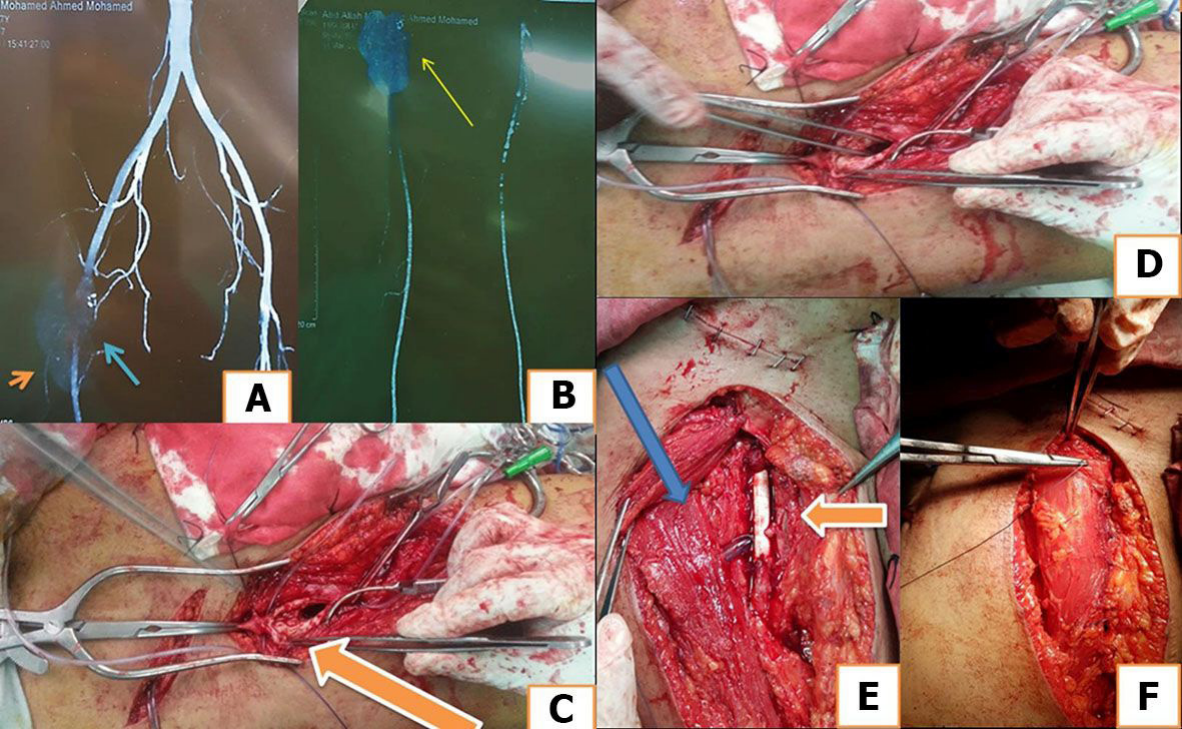
(A) Blue arrow indicates Behçet's pseudoaneurysm of the right common femoral artery involving the origin of the profunda femoris artery (orange arrow), seen on computed tomography angiography (CTA); (B) yellow arrow indicates size of the pseudo aneurysm (5x5cm); (C, D) orange arrow showing pseudoaneurysm opened after proximal and distal control, (E) orange arrow showing proximal and distal anastomoses with 6x60 cm PTFE graft and ligation of distal profunda femoris artery with Sartorius muscle flap (blue arrow); (F) showing closure of the wound after Sartorius muscle flap from same side.

There was no correlation between the type of procedure and the presentation or the size of the pseudoaneurysm (Figure 4).

### Follow-up

**Figure 5:**
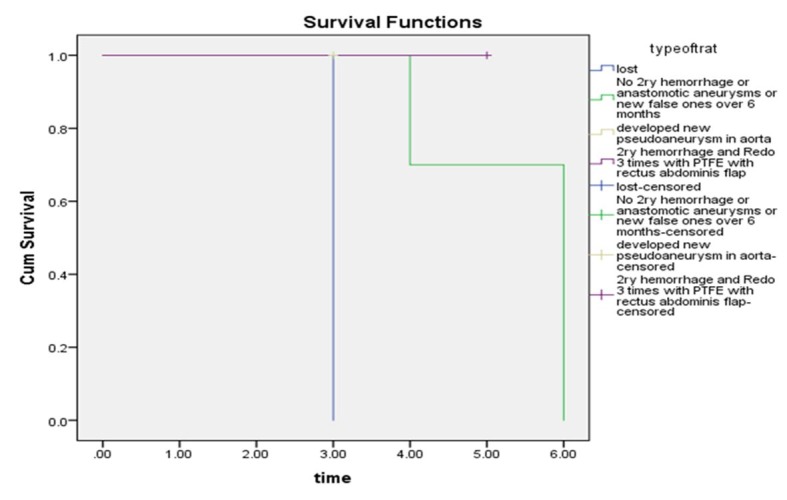
Follow-up over first 6 months.

The first six months of follow-up are illustrated in Figure 5.

## DISCUSSION

Arterial involvement reported in patients with BD is mostly of the major arteries in the form of pseudoaneurysms, aneurysms, occlusions, and thrombosis^5^^,^ 6 as was evidenced in our study.

Large vessels are involved in up to 40% of BD patients. Veins are more often involved than arteries.^6^

Peripheral arteries can be involved in BD, especially the SFA in the form of pseudoaneurysm.^6^^,^ 8

Affected histological features include: plasma cell infiltration, endothelial cell proliferation, perivascular lymphocytic infiltrate and disruption of elastic lamina, elimination of the tunica media, and vasculitis of the small vessels in vasa vasorum, which leads to stenoses, aneurysms, and thromboses in arteries and veins.^9^

As shown by the presence of acute and subacute DVT in some of the patients in this study, venous thrombosis may coexists with arterial lesions, as seen in many studies.^10^

We also recommend anticoagulation (subcutaneous enoxaparin) for deep venous thrombosis if associated with arterial aneurysm and surgical intervention is needed.

The patients in our study ranged in age from 30 to 40 years (mean, 33.8±2.6 years). The disease mainly affects young males of Middle Eastern and Far Eastern ancestry aged between 20 and 40 years.^9^

Behçet’s aneurysm has a tendency to affect multiple sites and can involve any artery.^11^

Management of peripheral artery aneurysm in BD is dependent on the site of the artery involved and on presentation, whether there is rupture or impending rupture and whether the disease is active or in remission phase.^12^

Higher doses in the initial period (pulse solumedrol 1 gm/ BID with monitoring blood pressure and blood glucose) were used with patients that responded well to therapy, and we did not notice any complications linked with prednisone treatment.

Venous thrombosis is a common complication of BD and may be associated with pulmonary emboli (fatal). Deep veins of the lower extremities are a very common DVT site. Anticoagulation added to colchicine can be used for treatment of venous thrombosis.^13^

The prevalence of deep venous thrombosis in the series was 26.6%.

In a previous study of 137 Turkish patients, reported incidence of venous and arterial involvement was 24% and 3% respectively.^14^ Japanese and European series reported higher incidences of arterial involvement.^15^^,^ 16 However, we believe that smaller sample size is a limitation of our study; larger numbers are needed to reach a conclusion about the exact incidence of venous lesions in Egyptian patients.

The traditional treatment for aneurysmal lesions in BD patients is surgical repair and grafting with either vein or synthetic materials. Graft occlusion, anastomotic pseudo-aneurysm formation or postoperative infection are reported,^6^ as unfortunately happened in one patient in our series with secondary hemorrhage occurring three times. This can be explained by disease progression, despite having performed anastomoses to apparently healthy segments. However, anatomopathological examination demonstrated focal inflammatory infiltrate (after the third repair) necessitating intraoperative frozen pathological examination to determine healthy arterial segments. Close follow-up with immunosuppressants may prevent arterial complications in BD patients.

Rates of success and fewer complications after surgery in patients with BD are affected by the timing of intervention (acute flare-up or the remission phase). Emergency interventions are done at any time, but the golden period to intervene is the remission period, when the vasculature is much more stable, not in an acute inflammation. High dose steroids should be given before intervention and continued afterwards in emergency cases only. This strategy may decrease the complication rate.^17^^-^ 20

## CONCLUSION

Surgical repair with muscle flap coverage with or without ligation of profunda femoris artery does not affect prognosis. There is a need for a new way to assess healthy segments rather than inspection. We suggest examination of a frozen section of the artery from the site of anastomosis, to reduce the risk of recurrence.
